# Associations of online religious participation during COVID-19 lockdown with subsequent health and well-being among UK adults

**DOI:** 10.1017/S0033291722000551

**Published:** 2023-07

**Authors:** Koichiro Shiba, Richard G. Cowden, Natasha Gonzalez, Yusuf Ransome, Atsushi Nakagomi, Ying Chen, Matthew T. Lee, Tyler J. VanderWeele, Daisy Fancourt

**Affiliations:** 1 Department of Epidemiology, Harvard T.H. Chan School of Public Health, Boston, MA, USA; 2 Human Flourishing Program, Institute for Quantitative Social Science, Harvard University, Cambridge, MA, USA; 3 Independent Researcher, Madrid, Spain; 4 Department of Social and Behavioral Sciences, Yale University School of Public Health, New Haven, CT, USA; 5 Department of Social Preventive Medical Sciences, Center for Preventive Medical Sciences, Chiba University, Chiba, Japan; 6 Department of Biostatistics, Harvard T.H. Chan School of Public Health, Boston, MA, USA; 7 Department of Behavioural Science and Health, University College London, London, UK

**Keywords:** COVID-19, health, longitudinal study, online communication, outcome-wide epidemiology, religion, religious service attendance, United Kingdom, well-being

## Abstract

**Background:**

In-person religious service attendance has been linked to favorable health and well-being outcomes. However, little research has examined whether online religious participation improves these outcomes, especially when in-person attendance is suspended.

**Methods:**

Using longitudinal data of 8951 UK adults, this study prospectively examined the association between frequency of online religious participation during the stringent lockdown in the UK (23 March –13 May 2020) and 21 indicators of psychological well-being, social well-being, pro-social/altruistic behaviors, psychological distress, and health behaviors. All analyses adjusted for baseline socio-demographic characteristics, pre-pandemic in-person religious service attendance, and prior values of the outcome variables whenever data were available. Bonferroni correction was used to correct for multiple testing.

**Results:**

Individuals with online religious participation of ≥1/week (*v.* those with no participation at all) during the lockdown had a lower prevalence of thoughts of self-harm in week 20 (odds ratio 0.24; 95% CI 0.09–0.62). Online religious participation of <1/week (*v.* no participation) was associated with higher life satisfaction (standardized *β* = 0.25; 0.11–0.39) and happiness (standardized *β* = 0.25; 0.08–0.42). However, there was little evidence for the associations between online religious participation and all other outcomes (e.g. depressive symptoms and anxiety).

**Conclusions:**

There was evidence that online religious participation during the lockdown was associated with some subsequent health and well-being outcomes. Future studies should examine mechanisms underlying the inconsistent results for online *v.* in-person religious service attendance and also use data from non-pandemic situations.

## Introduction

The goal of public health is to promote comprehensive health, including psychological, social, and spiritual well-being (Grad, 2002; UN General Assembly, 2015; VanderWeele, 2017a). Religion is principally oriented toward the promotion of spiritual well-being but can enhance other dimensions of health and well-being (Idler, 2014; Ransome, 2020; VanderWeele, 2017c). An integral component of many religious traditions is religious service attendance. Approximately 40% of the world's population attends religious services at least weekly (Pew, 2017). Growing evidence suggests that religious service attendance is associated with indices of favorable health and well-being, including reduced mortality and unhealthy behaviors (e.g. smoking), lower levels of psychological distress (e.g. depressive symptoms), and higher levels of psychosocial well-being (e.g. life satisfaction, social connectedness) across adulthood (Chen, Kim, & VanderWeele, 2021; Chen, Koh, Kawachi, Botticelli, & VanderWeele, 2020; Chen & VanderWeele, 2018; Li, Stampfer, Williams, & VanderWeele, 2016; Pawlikowski, Białowolski, Węziak-Białowolska, & VanderWeele, 2019).

The coronavirus disease 2019 (COVID-19) pandemic disrupted traditional forms of religious engagement; in-person religious services were often included in the non-essential activities suspended by public health measures (e.g. stay-at-home orders) to limit transmission of infection (Oxholm, Rivera, Schirrman, & Hoverd, 2021; Pew, 2020; Schuchat & CDC COVID-19 Response Team, 2020). Following public health experts' recommendations, many religious communities shifted congregational activities from in-person to online (World Health Organization, 2020). Although online religious participation could have health and well-being benefits comparable to those from in-person religious service attendance as there are shared features (e.g. content of services and opportunity for reflection), other important health-promoting features of in-person religious service attendance (e.g. communal religious practices and casual communication with fellow adherents that involves bodily co-presence) may not be adequately replaced by online participation (VanderWeele, 2020). Given the mass transition of faith-based communities to online forms and the potential continuation of online or hybrid worship during and after the COVID-19 pandemic, research is needed to identify the health-related implications of online religious participation. However, no study to date has rigorously examined the potential effects of online religious participation on health and well-being outcomes using longitudinal data.

In this large prospective study of UK adults, we examined the associations of online religious participation during a period of stringent lockdown in the UK (23 March to 13 May 2020) – when in-person religious services were suspended – with an array of subsequent health and well-being outcomes including psychological well-being, social well-being, pro-social/altruistic behaviors, psychological distress, and health behaviors.

## Methods

### Data

We used data from the UCL COVID-19 Social Study, a large prospective panel study on psychological and social experiences of UK adults (≥18 years old) during the COVID-19 pandemic. Multiple non-probability sampling strategies were used to obtain a non-random but well-stratified sample, including those from lower socioeconomic backgrounds and vulnerable groups (e.g. older adults, adults with pre-existing mental health conditions). All data were weighted to proportions of gender, age, ethnicity, education, and country of living obtained from the Office for National Statistics. Further details on sampling and weighting are available elsewhere (CSSUserGuide, 2021; Fancourt, Steptoe, & Bu, 2021).

**Fig. 1.**
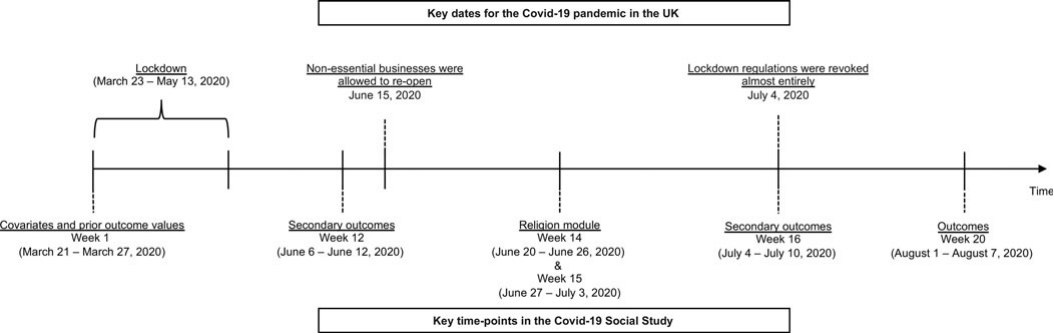
Timelines of data collection and key dates for the COVID-19 pandemic in the UK.

The study began on 21 March 2020 and collected data weekly from online participants. For this analysis, we used data from the participants recruited from 21 to 27 March 2020 (week 1: *n* = 28 847). Of those, 11 424 individuals completed the religion module administered in week 14 (20–26 June 2020) and week 15 (27 June–3 July 2020), which assessed the frequency of online religious participation. We excluded participants with missing survey weights (*n* = 49) and those who did not participate in the week 20 survey (1–7 August 2020), from which the outcome data were drawn (*n* = 2424). The final analytic sample consisted of 8951 individuals. Figure 1 illustrates the timeline of data collection and key dates for the COVID-19 pandemic in the UK.

### Measures

#### Online religious participation

The religion module was administered in weeks 14 and 15. Participants retrospectively reported the frequency of online religious service attendance during lockdown by answering the question, ‘During lockdown have you engaged in any online or digital religious activities such as watching streamed services, watching video prayers, joining online faith discussion groups, or receiving other digital spiritual support?’ from the options: 1 = more than once a week, 2 = once a week, 3 = two or three times a month, 4 = one or more times a year, and 5 = not at all. Consistent with previous research involving in-person service attendance (Chen et al., 2020), responses were grouped into three categories: ‘not at all’, ‘<1/week’, or ‘≥1/week’.

#### Outcomes

To reduce the possibility of recall bias for the online religious participation variable, we used the outcome data from week 20, so there was an approximately 1-month interval between the religion module and outcome assessment. We examined 21 indicators of health and well-being as outcomes, including psychological well-being (life satisfaction, happiness, and meaning), social well-being (social support and loneliness), pro-social/altruistic behaviors (volunteering, caring, compliance with social isolation, and compliance with social distancing), psychological distress (depressive symptoms, anxiety, number of minor stressors, number of major stressors, and thoughts of self-harm), and health behaviors (unhealthy change in smoking, unhealthy change in alcohol drinking, unhealthy change in diet, gentle physical activity, high-intensity physical activity, exercising at home, and good sleep). Online Supplementary Table S1 provides further details about each outcome measurement.

#### Covariates

All covariates were taken from week 1 (21–27 March 2020); the strict lockdown in the UK began on 23 March 2020. These covariates included sociodemographic factors (age, gender, race/ethnicity, living alone, education, employment, key worker role, low income), number of self-reported health conditions, frequency of service attendance before the pandemic, compliance with not leaving home, social relationships (frequency of meeting up with people in usual life, number of close friends), personality (neuroticism, extraversion, openness, agreeableness, conscientiousness), and baseline health behaviors (current smoking status, number of alcoholic drinks in the past week). To reduce the possibility of reverse causation (i.e. health and well-being affecting participation in online religious activities), we also adjusted for prior values of the outcomes. Specifically, adjustments were made for all week 20 outcome measures using the data provided in week 1, except for happiness, compliance with social distancing, thoughts of self-harm, and unhealthy change in diet, which were not measured in week 1 (CSSUserGuide, 2021).

### Statistical analysis

We used an outcome-wide analytic approach, which enables holistic assessment of the impact of a single exposure on a range of outcomes and has several methodological advantages (e.g. being less susceptible to p-hacking and publication bias) (VanderWeele, 2017b; VanderWeele, Mathur, & Chen, 2020). We used separate regression models to regress outcomes on online religious participation during lockdown, adjusting for both covariates measured at week 1 and prior outcome values wherever data were available. We used different models depending on the type of outcome: (1) linear regression for continuous outcomes (life satisfaction, happiness, meaning, social support, loneliness, compliance with social isolation, depressive symptoms, anxiety, number of minor and major stressors), (2) Poisson regression for non-rare binary outcomes with a prevalence ≥10% (compliance with social distancing, no unhealthy change in drinking, no unhealthy change in diet, gentle physical activity, high-intensity physical activity, exercising at home, good sleep), and (3) logistic regression for rare binary outcomes with a prevalence <10% (volunteering, caring, no unhealthy change in smoking behaviors, thoughts of self-harm). All continuous outcomes were standardized (mean = 0, s.d. = 1), so the effect estimates can be interpreted as standard deviation changes in the outcome variable. Poisson regression models for non-rare binary outcomes estimate risk ratios, and logistic regression models for rare binary outcomes estimate odds ratios approximating risk ratios. We used Bonferroni correction to account for multiple testing.

We performed three sensitivity analyses. First, to evaluate the robustness of our effect estimates to unmeasured confounding, we calculated *E* values for each exposure–outcome association (VanderWeele & Ding, 2017). *E* values quantify the minimum strength of association on the risk ratio scale that an unmeasured confounder would need to have with both the exposure and outcome, above and beyond the adjusted covariates, to explain away the observed association. Second, we performed subgroup analysis to examine whether associations between online religious participation and outcomes differ by frequency of in-person service attendance before the COVID-19 pandemic (‘Not at all’ and ‘<1/week or more often’). Third, we examined outcomes from week 16 (one week after the religion module) to examine the robustness of the results to the timing of outcome assessment.

We used multiple imputation by chained equations to impute missing data on all variables, using the *mice* R package (van Buuren & Groothuis-Oudshoorn, 2011). After generating five imputed datasets, we performed the analyses described above using each imputed dataset and combined the results across imputations. All analyses were conducted in R, version 3.6.0.

## Results

Table 1 shows baseline characteristics of the weighted study sample according to the frequency of online religious participation during the lockdown. Compared to those with no participation in online religious activities (*n* = 7795), individuals with participation of ⩾1/week (*n* = 542) reported higher psychological well-being (i.e. life satisfaction and meaning) at the beginning of the lockdown (week 1), but no apparent difference in psychological distress, such as depressive symptoms. On the other hand, individuals with participation of <1/week (*n* = 613) reported the lowest psychological well-being (e.g. mean life satisfaction = 5.32 for participation of <1/week *v.* 5.49 for no participation and 5.95 for participation of ≥1/week) and the highest baseline loneliness and psychological distress (e.g. mean depressive symptoms = 7.1 for participation of <1/week *v.* 6.1 for both no participation and participation of ≥1/week).

**Table 1.** Weighted sample characteristics at baseline by categories of online religious participation during the lockdown (*n* = 8951)

Characteristics at the beginning of lockdown	Online religious participation during lockdown					
	Not at all		<1/week		≥1/week	
	*n* (%)	Mean (s.d.)	*n* (%)	Mean (s.d.)	*n* (%)	Mean (s.d.)
Total	7795 (87.1)		613 (6.8)		542 (6.1)	
Sociodemographic factors						
Age, years		48 (16)		45 (19)		53 (17)
Female gender	3870 (49.6)		344 (63.3)		322 (52.5)	
Non-white ethnicity	892 (11.4)		128 (23.7)		123 (20.1)	
Living alone	1426 (18.3)		112 (20.6)		126 (20.6)	
Education						
GCSE or below	2603 (33.4)		141 (26.0)		176 (28.7)	
A levels or equivalent	2644 (33.9)		196 (36.2)		188 (30.6)	
Degree or above	2549 (32.7)		205 (37.8)		249 (40.7)	
Employed	4388 (56.3)		288 (53.1)		286 (46.7)	
Any key worker role	1654 (21.2)		126 (23.1)		88 (14.4)	
Low income (<£30 000)	3367 (48.4)		229 (50.7)		285 (53.5)	
Physical health						
Number of health conditions		0.92 (1.26)		0.81 (1.24)		1.06 (1.36)
Pre-pandemic service attendance						
At least once a week	41 (0.5)		47 (8.6)		425 (69.3)	
Less than once a week	1048 (13.4)		361 (66.5)		119 (19.4)	
Not at all	6707 (86.0)		135 (24.9)		70 (11.4)	
Not leaving home	4 073 (53.4)		302 (56.1)		357 (59.1)	
Social relationships						
Meeting up with people in usual life						
Every day	774 (9.9)		75 (13.8)		73 (11.9)	
Once a week or more often	4312 (55.3)		316 (58.2)		395 (64.5)	
Less than once a week	2710 (34.8)		152 (28.0)		145 (23.6)	
Number of close friends		4.3 (3.1)		5.0 (3.0)		5.4 (3.3)
Personality						
Neuroticism (range: 3–21)		11.4 (4.5)		12.3 (4.3)		11.4 (4.8)
Extraversion (range: 3–21)		12.2 (4.3)		12.6 (4.0)		12.6 (4.0)
Openness (range: 3–21)		14.6 (3.2)		14.8 (3.3)		14.6 (3.4)
Agreeableness (range: 3–21)		15.3 (3.2)		15.6 (2.9)		16.4 (2.9)
Conscientiousness (range: 3–21)		15.8 (2.9)		15.4 (2.8)		16.1 (2.8)
Prior psychological well-being						
Life satisfaction (range: 0–10)		5.49 (2.46)		5.32 (2.29)		5.95 (2.45)
Meaning (range: 0–10)		5.85 (2.71)		5.82 (2.52)		6.38 (2.60)
Prior social well-being						
Social support (range: 1–30)		22 (6)		22 (6)		23 (6)
Loneliness (range: 1–9)		4.80 (1.91)		5.06 (1.85)		4.73 (1.92)
Pro-social/altruistic behaviors						
Volunteering	378 (5.0)		64 (11.9)		42 (7.1)	
Caring	1354 (17.9)		90 (16.9)		121 (20.1)	
Compliance with social isolation (range: 1–7)		6.43 (0.90)		6.38 (0.79)		6.42 (0.94)
Prior psychological distress						
Depressive symptoms (range: 0–27)		6.1 (5.9)		7.1 (6.1)		6.1 (6.2)
Anxiety (range: 0–21)		5.6 (5.7)		6.9 (6.0)		5.5 (5.8)
Number of minor stressors (range: 0–16)		4.7 (3.0)		5.3 (3.1)		4.1 (2.8)
Number of major stressors (range: 0–16)		1.54 (2.04)		1.73 (2.19)		1.48 (2.09)
Prior health behaviors						
Smoking status						
Current smoker	851 (10.9)		63 (11.6)		23 (3.7)	
Ex-smoker	1983 (25.4)		99 (18.3)		100 (16.2)	
Non-smoker	4962 (63.6)		380 (70.1)		491 (80.0)	
Number of alcoholic drinks in the past week		3.7 (5.1)		3.2 (5.3)		2.7 (4.5)
No unhealthy change in smoking	7417 (96.3)		531 (98.0)		601 (98.9)	
No unhealthy change in alcohol drinking	6598 (85.3)		472 (87.1)		538 (88.6)	
Gentle physical activity	4505 (59.5)		350 (65.6)		411 (68.5)	
High-intensity physical activity	982 (13.0)		89 (16.8)		78 (13.2)	
Exercising at home	2467 (32.5)		203 (38.0)		182 (30.6)	
Good sleep	2956 (37.9)		144 (26.6)		247 (40.4)	

s.d., standard deviation.

Lockdown was imposed in the UK on 23 March 2020, and we used data from week 1 of the survey (21–27 March 2020). Data were weighted to the proportions of gender, age, ethnicity, education, and country of living obtained from the Office for National Statistics. Columns do not necessarily sum up exactly to 8951 because of rounding after weighting.

Table 2 shows estimated *β* coefficients (continuous outcomes), risk ratios (non-rare binary outcomes), and odds ratios (rare binary outcomes) for the online religious participation categories, adjusting for covariates. In week 20, those who participated in online religious activities <1/week had higher life satisfaction (standardized *β* = 0.25; 95% CI 0.11–0.39) and happiness (standardized *β* = 0.25; 0.08–0.42) than those with no participation. These associations remained below the *p* = 0.05 threshold after accounting for multiple testing via Bonferroni correction. Participation of ≥1/week (*v.* no participation at all) was associated with decreased thoughts of self-harm (odds ratio 0.24; 0.09–0.62). There was modest evidence of associations between participation of ≥1/week and increased compliance with social distancing (risk ratio = 1.08; 1.01–1.16), no unhealthy change in alcohol drinking in the past week (risk ratio = 1.08; 1.01–1.15), and increased loneliness (standardized *β* = 0.14; 0.01–0.26). However, none of these associations were below the *p* = 0.05 after Bonferroni correction. We found little evidence of associations between online religious participation of either frequency and other health and well-being outcomes.

**Table 2.** Online religious participation during lockdown and subsequent health and well-being in the UK (*n* = 8951)^a^

Outcomes in week 20	Online religious participation during lockdown				
	Not at all (*n* = 7795)	<1/week (*n* = 613)		⩾1/week (*n* = 542)	
	Reference	RR/OR/*β*^b^^,^^c^	95% CI	RR/OR/*β*^b^^,^^c^	95% CI
Psychological well-being					
Life satisfaction	0.00	0.25***	(0.11–0.39)	0.01	(−0.16 to 0.18)
Happiness	0.00	0.25***	(0.08–0.42)	−0.05	(−0.24 to 0.14)
Meaning	0.00	0.12	(−0.04 to 0.28)	−0.02	(−0.19 to 0.14)
Social well-being					
Social support	0.00	0.09	(−0.06 to 0.23)	−0.11	(−0.26 to 0.05)
Loneliness	0.00	−0.12	(−0.30 to 0.06)	0.14*	(0.01–0.26)
Pro-social/altruistic behaviors					
Volunteering	1.00	1.48	(0.80–2.75)	1.09	(0.48–2.49)
Caring	1.00	1.58	(0.98–2.56)	1.17	(0.62–2.21)
Compliance with social isolation	0.00	−0.17	(−0.52 to 0.17)	−0.08	(−0.37 to 0.21)
Compliance with social distancing	1.00	0.95	(0.84–1.08)	1.08*	(1.01–1.16)
Psychological distress					
Depressive symptoms	0.00	−0.09	(−0.28 to 0.10)	0.02	(−0.16 to 0.21)
Anxiety	0.00	−0.09	(−0.25 to 0.07)	0.04	(−0.14 to 0.22)
Number of minor stressors	0.00	0.06	(−0.11 to 0.24)	0.08	(−0.15 to 0.30)
Number of major stressors	0.00	−0.12	(−0.28 to 0.04)	0.22	(−0.16 to 0.61)
Thoughts of self-harm	1.00	1.17	(0.43–3.16)	0.24**	(0.09–0.62)
Health behaviors					
No unhealthy change in smoking	1.00	1.01	(0.31–3.32)	0.80	(0.22–2.96)
No unhealthy change in alcohol drinking	1.00	0.96	(0.85–1.09)	1.08*	(1.01–1.15)
No unhealthy change in diet	1.00	0.97	(0.86–1.09)	0.95	(0.84–1.07)
Gentle physical activity	1.00	0.84	(0.68–1.03)	1.06	(0.85–1.32)
High-intensity physical activity	1.00	0.84	(0.48–1.47)	1.15	(0.43–3.10)
Exercising at home	1.00	1.02	(0.76–1.38)	1.19	(0.85–1.67)
Good sleep	1.00	1.01	(0.78–1.31)	1.08	(0.87–1.34)

CI, confidence interval; RR, risk ratio; OR, odds ratio.

a Online religious participation during lockdown (23 March–13 May 2020) was assessed in the religion module conducted in week 14 (20–26 June 2020) and week 15 (27 June–3 July 2020). Outcomes were assessed in week 20 (1–7 August 2020). Covariates were measured at the beginning of the lockdown (week 1, 21–27 March 2020). The analytic sample was restricted to those who had participated in the religion module (weeks 14–15) and completed the survey in both weeks 1 and 20. Multiple imputation was performed to impute missing data on the covariates and the outcomes.

b All continuous outcomes (life satisfaction, happiness, meaning, social support, loneliness, compliance with social isolation, depressive symptoms, anxiety, and number of minor and major stressors) were standardized (mean = 0, standard deviation, 1), and *β* was the standardized effect size. The estimates for the outcomes of volunteering, caring, no unhealthy change in smoking behaviors, and thoughts of self-harm were odds ratios estimated via weighted logistic regression; these outcomes were rare (prevalence <10%), so the odds ratios would approximate the risk ratios. The estimates for other dichotomized outcomes (compliance with social distancing, no unhealthy change in drinking, no unhealthy change in diet, gentle physical activity, high-intensity physical activity, and good sleep) were risk ratios estimated via weighted Poisson regression.

c All models were controlled for participants' sociodemographic characteristics (age, gender, race, living alone, education, employment, any key worker role, and low income), health (number of health conditions, current smoking, and number of alcohol drinks in the past week), pre-pandemic religious service attendance, not leaving home, social relationships (frequency of meeting up with people in usual life and number of close friends), personality (neuroticism, extraversion, openness, agreeableness, and conscientiousness), and the prior outcome values wherever data were available. Specifically, the adjustment of the prior values were made for the following outcomes simultaneously: psychological well-being (life satisfaction and meaning), social well-being (social support and loneliness), pro-social/altruistic behaviors (volunteering, caring, and compliance with social isolation), psychological distress (depressive symptoms, anxiety, and number of major/minor stressors), health behaviors (change in smoking, change in alcohol drinking, gentle/high-intensity physical activity, exercising at home, and sleep). Data were weighted to the proportions of gender, age, ethnicity, education, and country of living obtained from the Office for National Statistics.

*p < 0.05 before Bonferroni correction; **p < 0.01 before Bonferroni correction; ***p < 0.05 after Bonferroni correction (the *p* value cutoff for Bonferroni correction is *p* = 0.05/21 outcomes = *p* < 0.0024).

The calculated *E* values (Table 3) suggested that some observed associations between online religious participation and subsequent well-being were moderately robust to an unmeasured confounder. For example, for the association between online religious participation of <1/week and life satisfaction (standardized *β* = 0.25), an unmeasured confounder would need to be associated with both the exposure and outcome – above and beyond the adjusted covariates – by a risk ratio of 1.82 to fully explain away the observed association and by 1.45-fold to shift the CI to include the null value. As shown in online Supplementary Table S2, the conditional associations of the observed covariates with outcomes were generally smaller than the *E* values for online religious participation, even for covariates with particularly strong associations with an outcome. For example, the risk ratio for the conditional association between life satisfaction and its prior value from week 1 was 1.17, whereas the *E* value for online participation of <1/week was 1.82.

**Table 3.** Robustness to unmeasured confounding (*E* values) of associations between online religious participation during lockdown and subsequent health and well-being (*n* = 8951)

Outcomes in week 20	Online religious participation during lockdown			
	<1/week (*n* = 613)		≥1/week (*n* = 542)	
	*E* value^a^			
	Effect estimate^b^	CI limit^c^	Effect estimate^b^	CI limit^c^
Psychological well-being				
Life satisfaction	1.82	1.45	1.11	1.00
Happiness	1.82	1.40	1.27	1.00
Meaning	1.47	1.00	1.16	1.00
Social well-being				
Social support	1.39	1.00	1.45	1.00
Loneliness	1.47	1.00	1.53	1.17
Pro-social/altruistic behaviors				
Volunteering	2.32	1.00	1.40	1.00
Caring	2.54	1.00	1.62	1.00
Compliance with social isolation	1.61	1.00	1.36	1.00
Compliance with social distancing	1.29	1.00	1.37	1.11
Psychological distress				
Depressive symptoms	1.39	1.00	1.16	1.00
Anxiety	1.39	1.00	1.23	1.00
Number of minor stressors	1.30	1.00	1.36	1.00
Number of major stressors	1.47	1.00	1.74	1.00
Thoughts of self-harm	1.62	1.00	7.80	2.61
Health behaviors				
No unhealthy change in smoking	1.11	1.00	1.81	1.00
No unhealthy change in alcohol drinking	1.25	1.00	1.37	1.11
No unhealthy change in diet	1.21	1.00	1.29	1.00
Gentle physical activity	1.67	1.00	1.31	1.00
High-intensity physical activity	1.67	1.00	1.57	1.00
Exercising at home	1.16	1.00	1.67	1.00
Good sleep	1.11	1.00	1.37	1.00

CI, confidence interval.

a See VanderWeele and Ding (2017) for the formula for calculating *E* values.

b *E* values for effect estimates are the minimum strength of association on the risk ratio scale that an unmeasured confounder would need to have with both the exposure and the outcome, above and beyond the measured covariates, to fully explain away the observed association of each level of online religious participation during lockdown (reference: ‘Not at all’) with various outcomes as shown in Table 2.

c *E* values for the 95% CI limit closest to the null denote the minimum strength of association on the risk ratio scale that an unmeasured confounder would need to have with both the exposure and the outcome, above and beyond the measured covariates, to shift the 95% CI to include the null value.

Subgroup analysis stratified by pre-pandemic in-person service attendance (online Supplementary Table S3) showed similar point estimates. However, some associations became null after stratifying (e.g. online participation of <1/week and happiness among those without pre-pandemic in-person service attendance). When we assessed outcomes from an earlier time point (week 16), we found similar trends for most outcomes (relative to week 20) but no associations with life satisfaction and happiness (online Supplementary Table S4).

## Discussion

In this longitudinal study of UK adults, we examined the associations of online religious participation during the initial strict lockdown due to the COVID-19 pandemic with subsequent health and well-being. Our main findings are threefold. First, the most frequent online religious participation (≥1/week) was associated with a lower prevalence of thoughts of self-harm compared to no online religious participation. Second, we found an association with higher psychological well-being (i.e. life satisfaction and happiness) for intermittent participation (<1/week), but not for most frequent participation (≥1/week), compared to no participation. Third, except for these two findings, there was little evidence that online religious participation was associated with other indices of subsequent health and well-being.

The observed association between the most frequent online participation and lower prevalence of thoughts of self-harm is consistent with prior evidence on in-person service attendance. For example, previous US-based studies found associations with lower rates of suicide and deaths of despair (deaths from suicides and drugs/alcohol use) for in-person participation of ≥1/week but not participation of <1/week (Chen et al., 2020; VanderWeele, Li, Tsai, & Kawachi, 2016). These associations could be explained by strong beliefs against self-harm and suicide that are part of religious values (Dervic et al., 2004; Koenig, Koenig, King, & Carson, 2012). Many religious traditions – including Christianity, which constitutes 38% of the entire UK population and 80% of religious individuals in the UK and 83% of this study sample (Curtice, Clery, Perry, Phillips, & Rahim, 2019) – explicitly prohibit self-injury and suicide because life is considered a gift from God and worth protection and care. Scholars have also hypothesized that religious service attendance could lower suicide risk by promoting hope, purpose, and meaning (Idler, 2014); however, our outcome-wide analysis did not provide evidence for the association between online religious participation and a single-item measure of meaning.

The inverse U-shaped relationship between frequencies of online religious participation and life satisfaction and happiness was inconsistent with evidence that the more frequent in-person service attendance is increasingly associated with greater psychological well-being (Chen et al., 2021; Chen & VanderWeele, 2018). There are at least three possible explanations for this inconsistency. First, of the individuals with the most frequent online religious participation (≥1/week), 69.3% had attended in-person religious services ≥1/week before the pandemic. These individuals with high levels of pre-pandemic religious engagement (*v.* individuals with less frequent or no pre-pandemic in-person service attendance) may have lost more resources due to the suspended in-person services during the lockdown, such as social support from a congregation. Online religious participation may not have sufficiently replaced resources to fulfill the increased need for those who participated most frequently during the lockdown (Holmgreen, Tirone, Gerhart, & Hobfoll, 2017). This theorizing is supported by the *increased* loneliness among people with the most frequent online participation (Table 2). Second, as shown in Table 1, individuals with online participation of <1/week had poorer baseline well-being at the beginning of the lockdown; hence, they may have had more room for improving life satisfaction and happiness. Third, evidence suggests that moderate social contact via telephone or video may be beneficial, but high levels of virtual communication can result in greater distress (Bu, Steptoe, Mak, & Fancourt, 2021; Twenge, Spitzberg, & Campbell, 2019).

Despite observed associations with decreased thoughts of self-harm and increased life satisfaction and happiness, online religious participation was not associated with other outcomes, which contrasts with the evidence linking in-person service attendance to a range of health and well-being outcomes (Chen et al., 2021; Chen & VanderWeele, 2018). We provide four potential explanations for our results. First, online communication cannot replicate some physical features of in-person social interactions (e.g. touching, hugging), which could reduce stress (Cohen, Janicki-Deverts, Turner, & Doyle, 2015; Jakubiak & Feeney, 2017). Second, online religious participation is unlikely to provide the same benefits as in-person services because sacred places often play a central role in inner religious/spiritual experiences (Counted, Neff, Captari, & Cowden, 2020; Fancourt & Steptoe, 2019; Mazumdar & Mazumdar, 2004). Third, online services may not have provided the same quality of faith teachings and communal religious/spiritual experiences as in-person services. Fourth, evidence suggests that social support tends to be less beneficial when provided by people experiencing the same stressors (Rueger, Malecki, Pyun, Aycock, & Coyle, 2016). The helpfulness of online religious participation in our study may have been affected by the widespread impact of the COVID-19 pandemic. Indeed, the sensitivity analyses examining outcomes from earlier time points – times when stressors were likely more severe for many because some lockdown regulations were still imposed – indicated that the associations between online religious participation and psychological well-being were null.

The frequency of pre-pandemic religious service attendance in this study population was somewhat low (22.8% reported participation), given that 38% of the UK population identified themselves as Christians. However, the proportion of people who attend religious services in the UK has been reported to be smaller than the proportion of religious individuals in the same population, according to the British Social Attitudes survey – a survey of a representative sample of adults ≥18 years old in the UK. In 2018, approximately 11% of the UK population (*v.* 5.7% in our sample) attended a religious service at least once a week, 20% (*v.* 17.1% in our sample) reported more infrequent participation, and 66% reported no attendance (*v.* 77.2% in our sample) (Curtice et al., 2019). Because the UK's religious population has been decreasing over time, the even more infrequent self-reported religious service attendance in our study sample may be due to the time difference between the British Social Attitudes survey (2018) and our study (2020).

There were notable changes in the frequency when comparing pre-pandemic in-person service attendance and online attendance during the lockdown. Specifically, there was an increase in never attendees (77.2% before the pandemic to 87.1% during the lockdown) and frequent attenders (5.7–6.9%), while infrequent attendees decreased (17.1–6.1%). Although speculative, there are three potential explanations. First, the shift from in-person religious services to online services can cause both an increase and decrease in the frequency of service attendance. For example, the online format might have facilitated attendance among some people by removing barriers (e.g. not having to spend time to go to churches). At the same time, some people may have become less interested in service attendance because the online format does not come with the joy of social interactions with peers that they used to gain from in-person attendance or has some technical complexities (e.g. people not knowing how to attend services via the Internet). This might explain the increase in most frequent participation and no participation and decrease in infrequent participation. Secondly, some individuals who infrequently or never attended religious services before the pandemic may have started to seek opportunities to cope with the hardship/anxiety they experienced during the pandemic, which may explain the increase in most frequent service attendance during the lockdown. This is a form of reverse causation (i.e. health/well-being leading to more religious participation rather than service attendance causally affecting people's health/well-being), which we partly addressed by adjusting for outcome values at the beginning of the lockdown in the outcome-wide regression analysis. Third, while the infrequent attendance category (<1/week) can include a wide range of frequencies (ranging from, e.g. once every other week to once per year), online attendance was evaluated for a narrower timeframe of only 2 months (i.e. the hard lockdown between March and May 2020). Therefore, the category of infrequent online attendance during the lockdown could only catch a much smaller range of frequency (the least possible frequency was once over the 2 months). In other words, many individuals who would have been categorized as infrequent attenders if a wider timeframe were given (e.g. once per year) ended up being categorized as never attenders.

We note six limitations of the current study. First, frequency of online religious participation during the strict lockdown was retrospectively reported approximately 5 weeks after the lockdown ended on 13 May 2020, making the measurement susceptible to recall bias. We addressed this issue, at least partly, by obtaining the primary outcome information from the study wave conducted one month after measuring online religious participation. Second, we cannot preclude the possibility of unmeasured confounders; however, we leveraged the panel structure of the data and adjusted for a rich set of covariates, including prior levels of outcomes and pre-pandemic in-person religious service attendance. Moreover, the sensitivity analysis suggested robustness of the observed associations to residual confounding, at least for thoughts of self-harm as an outcome. Third, the earliest experience of online religious participation (i.e. exposure) may have preceded the pandemic or the covariate measurement in the first week of the hard lockdown. If one participated in online religious service before the pandemic, there will be residual confounding because we did not measure pre-pandemic online service attendance; however, we adjusted for a series of covariates, including pre-pandemic in-person attendance, which is likely correlated with online attendance strongly. If the covariate measurement was after the exposure, the models effectively adjust for mediators rather than confounders, and our results would be conservative estimates of the total effects of online religious participation. Fourth, some associations between online religious participation and the outcomes may be due to post-lockdown in-person service attendance rather than online participation because the outcomes in the main analysis were taken after lockdown regulations were revoked almost entirely. Fifth, there was attrition from the original participants in week 1 (*n* = 28 847) to the analytic sample (*n* = 8951). We conducted an *ad hoc* analysis comparing weighted characteristics in week 1 of these two samples and confirmed that the two samples had comparable distributions of the observed covariates (online Supplementary Table S5). However, we cannot eliminate the possibility that unmeasured factors might be associated with both the attrition and the outcomes. If the attrition is also associated with the exposure (i.e. online religious participation), the attrition may result in selection bias and induce non-causal associations (Hernán, Hernández-Díaz, & Robins, 2004). Lastly, the generalizability and transportability of the findings are limited. The sample was from an Internet-based study and not representative of the UK general population, although we used survey weights to make the estimates applicable to the wider population. Moreover, because the sample was from the UK population, 83% of the religious participants were Christian; thus, the results may not be transportable to other populations or religious groups. The results should be interpreted in the context of the pandemic and resulting lockdown because online religious participation may have differential impacts during non-crisis times when societies as a whole experience less distress.

In conclusion, there was only limited evidence that online religious participation during the lockdown promoted health and well-being among adults in the UK. To explore whether online religious participation has the potential to promote the same health and well-being benefits as in-person participation, future studies should investigate mechanisms underlying the weaker associations with the outcomes for online religious participation compared to prior findings regarding in-person service attendance. Research with data from non-pandemic situations is also needed to examine the benefits of online religious participation under less stressful conditions.

## Supplementary material

For supplementary material accompanying this paper visit https://doi.org/10.1017/S0033291722000551. click here to view supplementary material
