# Supplementary material for: Associations of online religious participation during COVID-19 lockdown with subsequent health and well-being among UK adults
Source: Psychol Med. 2022 Feb 22;53(9):3887–96. doi: 10.1017/S0033291722000551 (PMC10317791; doi:10.1017/S0033291722000551)
Supplement: Supplementary file 1 [file S0033291722000551sup001.docx]

**Online-Only Supplements for:**

Associations of online religious participation during COVID-19 lockdown with subsequent health and well-being among UK Adults

# Koichiro Shiba, PhD; Richard G. Cowden, PhD; Natasha Gonzalez, AB; Yusuf Ransome, DrPH; Atsushi Nakagomi, PhD; Ying Chen, ScD; Matthew T. Lee, PhD; Tyler J. VanderWeele, PhD; Daisy Fancourt, PhD

This document includes:

- Supplementary Table S1. List of outcome variables
- Supplementary Table S2. Baseline characteristics most strongly associated with health and well-being outcomes (n = 8 951)
- Supplementary Table S3. Online religious participation during lockdown and subsequent health and well-being in the UK by service attendance before the COVID-19 pandemic, (n = 8 951)
- Supplementary Table S4. Online religious participation during lockdown and subsequent health and well-being in the UK (n = 8 296)

Supplementary Table S1. List of outcome variables

| **Outcomes by Theme** | **Variable Name** | **Variable Type** | **Item Question** |
| --- | --- | --- | --- |
| 1. Psychological well-being | | | |
| Life satisfaction | onssat | Continuous (range: 0-10); higher values indicate greater life satisfaction. | Overall, in the past week, how satisfied have you been with your life? |
| Happiness | onshappy | Continuous (range: 0-10); higher values indicate greater happiness. | In the past week, how happy did you feel? |
| Meaning | onsworth | Continuous (range: 0-10); higher values indicate greater sense of meaning. | In the past week, to what extent have you felt the things you are doing in your life are worthwhile? |
| 2. Social well-being | | | |
| Social support | support | Continuous (range: 1-30); higher values indicate more support. | Brief Perceived Social Support Questionnaire (F-SozU K-6) |
| Loneliness | lonely | Continuous (range: 1-9); higher values indicate greater loneliness. | UCLA 3-item Loneliness Scale |
| 3. Pro-social/altruistic behaviors | | | |
| Volunteering | acta8 | Binary (0 = Did not do vs 1 = <30 mins/30 mins-2 hours/3-5 hours/6-+ hours) | How long you’ve spent doing different activities on a single day (the last week day): Volunteering |
| Caring | acta7 | Binary (0 = Did not do vs 1 = <30 mins/30 mins-2 hours/3-5 hours/6-+ hours) | How long you’ve spent doing different activities on a single day (the last week day): Caring for a friend or relative |
| Compliance with social distancing | socdist | Binary (0 = Not always/Not at all vs 1 = Yes, completely/Yes, to a large extent/Not applicable - I have not met with others or left my home in the last week) | When you go out or meet with others have you been maintaining social distancing? |
| Compliance with social isolation | followingisolation | Continuous (range: 1-7); higher values indicate greater compliance. | Are you following the recommendations from authorities to prevent spread of Covid-19? |
| 4. Psychological distress | | | |
| Depressive Symptoms | PHQ | Continuous (range:0-27); higher values indicate more depressive symptoms. | Patient Health Questionnaire-9 |
| Anxiety | GAD | Continuous (range: 0-21); higher values indicate more anxiety. | Generalized Anxiety Disorder-7 |
| Number of minor stressors | stressorsminor___1~  stressorsminor___16 | Continuous (total number of stressors; range: 0 -16) | Over the past week, have any of the following been worrying you at all, even if only in a minor way? |
| Number of major stressors | stressorsmajor___1~  stressorsmajor___16 | Continuous (total number of stressors; range: 0 -16) | Have any of these things been causing you SIGNIFICANT stress? (e.g. they have been constantly on your mind or have been keeping you awake at night) |
| Thoughts of self-harm | harm1 | Binary (0 = Not at all vs 1 = One or two days/more than half the days/nearly every day) | Over the last week, how often have been bothered by: Self-harming or deliberately hurting yourself |
| 5. Health behavior | | | |
| No unhealthy change in smoking | smokechange | Binary (0 = Less than usual/About the same/I don’t smoke vs 1 = More than usual) | Over the past week have you smoked more than usual? |
| No unhealthy change in alcohol drinking | alcoholchange | Binary (0 = Less than usual/About the same/I don’t drink alcohol vs 1 = More than usual) | Over the past week have you drunk alcohol more than usual? |
| No unhealthy change in diet | dietchange_3 | Binary (0 = About the same healthiness as usual/more healthy than usual vs 1 = Less healthy than usual) | Over the past week how has your diet been? |
| Gentle physical activity | actb3 | Binary (0 = Did not do vs 1 = <30 mins/30 mins-2 hours/3-5 hours/6-+ hours) | How long you’ve spent doing different activities on a single day (the last week day): Going out for a walk or other gentle physical activity |
| High intensity physical activity | actb4 | Binary (0 = Did not do vs 1 = <30 mins/30 mins-2 hours/3-5 hours/6-+ hours) | How long you’ve spent doing different activities on a single day (the last week day): Going out for moderate or high intensity activity (e.g, running, cycling or swimming) |
| Exercising at home | actb6 | Binary (0 = Did not do vs 1 = <30 mins/30 mins-2 hours/3-5 hours/6-+ hours) | How long you’ve spent doing different activities on a single day (the last week day): Exercising inside your home or garden (e.g. doing yoga, weights or indoor exercise) |
| Good sleep | sleep | Binary (0 = Average/Not good/Very poor vs 1 = Very good/Good) | Over the past week, how has your sleep been? |

Supplementary Table S2. Baseline characteristics most strongly associated with health and well-being outcomes (n = 8 951)^a^

| **Outcomes in week 20** | **Baseline characteristics from week 1 most strongly associated with the outcomes^b^** | | | | | |
| --- | --- | --- | --- | --- | --- | --- |
|  | **Top 1** | | **Top 2** | | **Top 3** | |
|  | **Variable** | **Est.^c^** | **Variable** | **Est.^c^** | **Variable** | **Est.^c^** |
| Psychological well-being |  |  |  |  |  |  |
| Life satisfaction | No unhealthy change in smoking | 1.19 | Meaning | 1.19 | Life satisfaction | 1.17 |
| Happiness | Meaning | 1.19 | Depressive symptoms | 1.18 | Social support | 1.15 |
| Meaning | Meaning | 1.26 | No unhealthy change in smoking | 1.18 | Depressive symptoms | 1.15 |
| Social well-being |  |  |  |  |  |  |
| Social support | Social support | 1.89 | No unhealthy change in smoking | 1.38 | Living alone | 1.36 |
| Loneliness | Loneliness | 1.75 | Depressive symptoms | 1.14 | Pre-pandemic service attendance: Not at all (vs. at least once a week) | 1.11 |
| Pro-social/altruistic behaviors |  |  |  |  |  |  |
| Volunteering | Volunteering | 14.2 | No unhealthy change in smoking | 3.08 | Meeting up with people in usual life: Less than once a week (vs. every day) | 2.62 |
| Caring | Caring | 12.7 | Smoking status: Non-smoker (vs. current smoker) | 1.61 | Smoking status: Ex-smoker (vs. current smoker) | 1.59 |
| Compliance with social isolation | Compliance with social isolation | 1.46 | Education: Degree or above (vs. GCSE or below) | 1.30 | High intensity physical activity | 1.20 |
| Compliance with social distancing | Pre-pandemic service attendance: Not at all (vs. at least once a week) | 1.12 | High intensity physical activity | 1.11 | Smoking status: Ex-smoker (vs. current smoker) | 1.10 |
| Psychological distress |  |  |  |  |  |  |
| Depressive symptoms | Depressive symptoms | 1.53 | Non-white ethnicity | 1.20 | Loneliness | 1.18 |
| Anxiety | Anxiety | 1.30 | Depressive symptoms | 1.21 | No unhealthy change in alcohol drinking | 1.15 |
| Number of minor stressors | Number of minor stressors | 1.34 | No unhealthy change in smoking | 1.20 | Number of major stressors | 1.12 |
| Number of major stressors | Number of major stressors | 1.30 | No unhealthy change in smoking | 1.26 | Pre-pandemic service attendance: Not at all (vs. at least once a week) | 1.19 |
| Thoughts of self-harm | Low income | 3.23 | Pre-pandemic service attendance: Not at all (vs. at least once a week) | 2.80 | Exercising at home | 2.31 |
| Health behaviors |  |  |  |  |  |  |
| No unhealthy change in smoking | Smoking status: Non-smoker (vs. current smoker) | 151 | Smoking status: Ex-smoker (vs. current smoker) | 13.9 | No unhealthy change in smoking | 2.20 |
| No unhealthy change in alcohol drinking | No unhealthy change in alcohol drinking | 1.12 | Non-white ethnicity | 1.08 | Pre-pandemic service attendance: Not at all (vs. at least once a week) | 1.07 |
| No unhealthy change in diet | Meeting up with people in usual life: Less than once a week (vs. every day) | 1.15 | Meeting up with people in usual life: Once a week or more (vs. every day) | 1.13 | Pre-pandemic service attendance: Less than once a week (vs. at least once a week) | 1.10 |
| Gentle physical activity | Gentile physical activity | 2.01 | Non-white ethnicity | 1.20 | Meeting up with people in usual life: Less than once a week (vs. every day) | 1.18 |
| High intensity physical activity | High intensity physical activity | 3.59 | Pre-pandemic service attendance: Less than once a week (vs. at least once a week) | 1.56 | Education: Degree or above (vs. GCSE or below) | 1.53 |
| Exercising at home | Exercising at home | 3.73 | Education: Degree or above (vs. GCSE or below) | 1.32 | Any key worker role | 1.31 |
| Good sleep | Good sleep | 2.32 | No unhealthy change in smoking | 1.58 | Depressive symptoms | 1.24 |

^a^ Online religious participation during lockdown (March 23 - May 13, 2020) was assessed in the religion module conducted in week 14 (June 20 - June 26, 2020) and week 15 (June 27 - July 3, 2020). Outcomes were assessed in week 20 (August 1 - August 7, 2020). Covariates were measured at the beginning of the lockdown (week 1, March 21 - March 27, 2020). The analytic sample was restricted to those who had participated in the religion module (weeks 14-15) and completed the survey in both weeks 1 and 20. Multiple imputation was performed to impute missing data on the covariates and the outcomes.

^b^ All models controlled for participants’ sociodemographic characteristics (age, gender, race, living alone, education, employment, any key worker role, and low income), health (number of health conditions, current smoking, and number of alcohol drinks in the past week), pre-pandemic religious service attendance, not leaving home, social relationships (frequency of meeting up with people in usual life and number of close friends), personality (neuroticism, extraversion, openness, agreeableness, and conscientiousness), and the prior outcome values wherever data were available. Specifically the adjustment of the prior values were made for the following outcomes simultaneously: psychological well-being (life satisfaction and meaning), social well-being (social support and loneliness), pro-social/altruistic behaviors (volunteering, caring, and compliance with social isolation), psychological distress (depressive symptoms, anxiety, and number of major/minor stressors), health behaviors (change in smoking, change in alcohol drinking, gentle/high intensity physical activity, exercising at home, and sleep). Data were weighted to the proportions of gender, age, ethnicity, education, and country of living obtained from the Office for National Statistics.

^c^ Estimates are on the risk ratio scale. For the continuous outcomes (life satisfaction, happiness, meaning, social support, loneliness, compliance with social isolation, depressive symptoms, anxiety, and number of minor and major stressors), we used the conversion outlined in VanderWeele and Ding (2017) to get an approximated risk ratio from a regression coefficient. For the rare binary outcomes (volunteering, caring, no unhealthy change in smoking behaviors, and thoughts of self-harm), the estimated odds ratios approximate risk ratios.

Supplementary Table S3. Online religious participation during lockdown and subsequent health and well-being in the UK by service attendance before the COVID-19 pandemic (n = 8 951)^a^

|  |  | **Service attendance before the pandemic** | | | | | | | | |
| --- | --- | --- | --- | --- | --- | --- | --- | --- | --- | --- |
| **Outcomes in week 20** |  | **Not at all**  **(n = 6 912)** | | | |  | **< 1/week or more often**  **(n = 2 039)** | | | |
|  |  | **Online religious participation during lockdown** | | | | | | | | |
|  | **Not at all** | **< 1/week**  **(n = 135)** | | $\boldsymbol{\geq}$ **1/week**  **(n = 70)** | |  | **< 1/week**  **(n = 408)** | | $\boldsymbol{\geq}$ **1/week**  **(n = 544)** | |
|  | **Reference** | **RR/OR/β^b,c^** | **95% CI** | **RR/OR/β** | **95% CI** |  | **RR/OR/β** | **95% CI** | **RR/OR/β** | **95% CI** |
| Psychological well-being |  |  |  |  |  |  |  |  |  |  |
| Life satisfaction | 0.00 | 0.30* | (0.03, 0.57) | 0.07 | (-0.26, 0.39) |  | 0.20** | (0.07, 0.33) | 0.09 | (-0.05, 0.23) |
| Happiness | 0.00 | 0.23 | (-0.07, 0.53) | 0.11 | (-0.21, 0.43) |  | 0.25*** | (0.09, 0.41) | 0.02 | (-0.15, 0.20) |
| Meaning | 0.00 | 0.21 | (-0.02, 0.45) | 0.10 | (-0.19, 0.39) |  | 0.10 | (-0.06, 0.26) | 0.06 | (-0.09, 0.22) |
| Social well-being |  |  |  |  |  |  |  |  |  |  |
| Social support | 0.00 | 0.03 | (-0.20, 0.27) | -0.18 | (-0.59, 0.22) |  | 0.07 | (-0.07, 0.21) | -0.02 | (-0.13, 0.10) |
| Loneliness | 0.00 | 0.02 | (-0.23, 0.27) | 0.21 | (-0.01, 0.43) |  | -0.16* | (-0.32, 0.00) | 0.03 | (-0.10, 0.16) |
| Pro-social/altruistic behaviors |  |  |  |  |  |  |  |  |  |  |
| Volunteering | 1.00 | 1.14 | (0.40, 3.22) | 1.46 | (0.22, 9.51) |  | 1.43 | (0.73, 2.78) | 1.24 | (0.65, 2.36) |
| Caring | 1.00 | 2.06 | (0.88, 4.82) | 1.98 | (0.70, 5.61) |  | 1.19 | (0.70, 2.02) | 0.85 | (0.52, 1.38) |
| Compliance with social isolation | 0.00 | -0.13 | (-0.52, 0.25) | -0.49 | (-1.27, 0.30) |  | -0.12 | (-0.40, 0.16) | 0.04 | (-0.16, 0.23) |
| Compliance with social distancing | 1.00 | 0.94 | (0.79, 1.12) | 1.12* | (1.03, 1.22) |  | 0.96 | (0.85, 1.08) | 1.02 | (0.95, 1.10) |
| Psychological distress |  |  |  |  |  |  |  |  |  |  |
| Depressive symptoms | 0.00 | -0.03 | (-0.32, 0.26) | 0.22 | (-0.1, 0.54) |  | -0.08 | (-0.26, 0.09) | -0.09 | (-0.21, 0.04) |
| Anxiety | 0.00 | -0.02 | (-0.3, 0.26) | 0.27 | (-0.21, 0.74) |  | -0.10 | (-0.25, 0.04) | -0.10 | (-0.20, 0.01) |
| Number of minor stressors | 0.00 | 0.01 | (-0.24, 0.25) | 0.36 | (-0.21, 0.93) |  | 0.04 | (-0.10, 0.18) | -0.02 | (-0.13, 0.08) |
| Number of major stressors | 0.00 | -0.12 | (-0.37, 0.13) | 0.98 | (-0.14, 2.11) |  | -0.11 | (-0.25, 0.02) | -0.06 | (-0.16, 0.04) |
| Thoughts of self-harm | 1.00 | 0.35 | (0.05, 2.30) | 0.00*** | (0.00, 0.00) |  | 1.98 | (0.71, 5.52) | 0.51 | (0.12, 2.11) |
| Health behaviors |  |  |  |  |  |  |  |  |  |  |
| No unhealthy change in smoking | 1.00 | 1.10 | (0.25, 4.84) | 0.17* | (0.04, 0.68) |  | 1.59 | (0.22, 11.6) | 2.53 | (0.56, 11.4) |
| No unhealthy change in alcohol drinking | 1.00 | 1.10* | (1.01, 1.19) | 1.09** | (1.03, 1.15) |  | 0.93 | (0.83, 1.05) | 1.05 | (0.99, 1.11) |
| No unhealthy change in diet | 1.00 | 0.96 | (0.81, 1.13) | 0.78 | (0.54, 1.11) |  | 1.00 | (0.89, 1.13) | 1.03 | (0.95, 1.11) |
| Gentle physical activity | 1.00 | 1.23** | (1.05, 1.44) | 1.37 | (0.94, 2.01) |  | 0.70*** | (0.57, 0.87) | 0.91 | (0.79, 1.05) |
| High intensity physical activity | 1.00 | 1.01 | (0.25, 3.97) | 0.48 | (0.17, 1.36) |  | 0.77 | (0.47, 1.25) | 0.89 | (0.61, 1.31) |
| Exercising at home | 1.00 | 1.28 | (0.84, 1.95) | 1.97** | (1.21, 3.20) |  | 0.94 | (0.70, 1.25) | 0.85 | (0.66, 1.10) |
| Good sleep | 1.00 | 1.29 | (0.79, 2.09) | 1.29 | (0.82, 2.03) |  | 0.92 | (0.71, 1.19) | 1.00 | (0.81, 1.23) |

Abbreviations: CI, confidence interval; RR, risk ratio; OR, odds ratio

* p<0.05 before Bonferroni correction; ** p<0.01 before Bonferroni correction; *** p<0.05 after Bonferroni correction (the p-value cutoff for Bonferroni correction is p = 0.05/21 outcomes = p <0.0024)

^a^ Online religious participation during lockdown (March 23 - May 13, 2020) was assessed in the religion module conducted in week 14 (June 20 - June 26, 2020) and week 15 (June 27 - July 3, 2020). Outcomes were assessed in week 20 (August 1 - August 7, 2020). Covariates were measured at the beginning of the lockdown (week 1, March 21 - March 27, 2020). The analytic sample was restricted to those who had participated in the religion module (weeks 14-15) and completed the survey in both weeks 1 and 20. Multiple imputation was performed to impute missing data on the covariates and the outcomes.

^b^ All continuous outcomes (life satisfaction, happiness, meaning, social support, loneliness, compliance with social isolation, depressive symptoms, anxiety, and number of minor and major stressors) were standardized (mean = 0, standard deviation, 1), and β was the standardized effect size. The estimates for the outcomes of volunteering, caring, no unhealthy change in smoking behaviors, and thoughts of self-harm were odds ratios estimated via weighted logistic regression; these outcomes were rare (prevalence <10%), so the odds ratios would approximate the risk ratios. The estimates for other dichotomized outcomes (compliance with social distancing, no unhealthy change in drinking, no unhealthy change in diet, gentle physical activity, high intensity physical activity, and good sleep) were risk ratios estimated via weighted Poisson regression.

^c^ All models controlled for participants’ sociodemographic characteristics (age, gender, race, living alone, education, employment, any key worker role, and low income), health (number of health conditions, current smoking, and number of alcohol drinks in the past week), pre-pandemic religious service attendance, not leaving home, social relationships (frequency of meeting up with people in usual life and number of close friends), personality (neuroticism, extraversion, openness, agreeableness, and conscientiousness), and the prior outcome values wherever data were available. Specifically, the adjustment of the prior values were made for the following outcomes simultaneously: psychological well-being (life satisfaction and meaning), social well-being (social support and loneliness), pro-social/altruistic behaviors (volunteering, caring, and compliance with social isolation), psychological distress (depressive symptoms, anxiety, and number of major/minor stressors), health behaviors (change in smoking, change in alcohol drinking, gentle/high intensity physical activity, exercising at home, and sleep). Data were weighted to the proportions of gender, age, ethnicity, education, and country of living obtained from the Office for National Statistics.

Supplementary Table S4. Online religious participation during lockdown and subsequent health and well-being in the UK (n = 8 296)^a^

| **Outcomes in week 16** | **Online religious participation during lockdown** | | | | | |
| --- | --- | --- | --- | --- | --- | --- |
|  | **Not at all**  **(n = 7 141)** | **< 1/week**  **(n = 498)** | | $\boldsymbol{\geq}$ **1/week**  **(n = 571)** | |  |
|  | **Reference** | **RR/OR/β^b,c^** | **95% CI** | **RR/OR/β^b,c^** | **95% CI** | |
| Psychological well-being |  |  |  |  |  | |
| Life satisfaction | 0.00 | 0.04 | (-0.17, 0.24) | -0.02 | (-0.21, 0.17) | |
| Happiness | 0.00 | 0.03 | (-0.23, 0.29) | -0.09 | (-0.29, 0.12) | |
| Meaning | 0.00 | 0.03 | (-0.15, 0.20) | -0.14 | (-0.35, 0.06) | |
| Social well-being |  |  |  |  |  | |
| Social support | 0.00 | 0.08 | (-0.07, 0.24) | -0.18* | (-0.32, -0.04) | |
| Loneliness | 0.00 | -0.24* | (-0.43, -0.05) | 0.06 | (-0.08, 0.20) | |
| Pro-social/altruistic behaviors |  |  |  |  |  | |
| Volunteering | 1.00 | 1.10 | (0.55, 2.22) | 2.41 | (0.86, 6.76) | |
| Caring | 1.00 | 1.45 | (0.84, 2.52) | 0.75 | (0.38, 1.48) | |
| Compliance with social isolation | 0.00 | -0.02 | (-0.33, 0.29) | 0.06 | (-0.26, 0.38) | |
| Compliance with social distancing | 1.00 | 0.99 | (0.89, 1.11) | 1.06 | (0.99, 1.13) | |
| Psychological distress |  |  |  |  |  | |
| Depressive symptoms | 0.00 | -0.13 | (-0.32, 0.06) | -0.03 | (-0.18, 0.12) | |
| Anxiety | 0.00 | -0.06 | (-0.23, 0.10) | -0.04 | (-0.19, 0.10) | |
| Number of minor stressors | 0.00 | -0.09 | (-0.27, 0.09) | 0.09 | (-0.15, 0.34) | |
| Number of major stressors | 0.00 | -0.15* | (-0.31, 0.00) | 0.11 | (-0.26, 0.48) | |
| Thoughts of self-harm | 1.00 | 0.62 | (0.19, 2.01) | 0.16*** | (0.06, 0.42) | |
| Health behaviors |  |  |  |  |  | |
| No unhealthy change in smoking | 1.00 | 0.62 | (0.17, 2.22) | 1.08 | (0.25, 4.67) | |
| No unhealthy change in alcohol drinking | 1.00 | 1.03 | (0.97, 1.10) | 1.01 | (0.93, 1.09) | |
| No unhealthy change in diet | 1.00 | 0.92 | (0.77, 1.11) | 1.01 | (0.89, 1.15) | |
| Gentle physical activity | 1.00 | 0.84 | (0.68, 1.03) | 0.92 | (0.75, 1.12) | |
| High intensity physical activity | 1.00 | 0.83 | (0.47, 1.47) | 1.26 | (0.49, 3.26) | |
| Exercising at home | 1.00 | 0.82 | (0.61, 1.09) | 1.03 | (0.75, 1.40) | |
| Good sleep | 1.00 | 1.17 | (0.89, 1.53) | 1.00 | (0.75, 1.33) | |

Abbreviations: CI, confidence interval; RR, risk ratio; OR, odds ratio

* p<0.05 before Bonferroni correction; *** p<0.05 after Bonferroni correction (the p-value cutoff for Bonferroni correction is p = 0.05/21 outcomes = p <0.0024)

^a^ Online religious participation during lockdown (March 23 - May 13, 2020) was assessed in the religion module conducted in week 14 (June 20 - June 26, 2020) and week 15 (June 27 - July 3, 2020). Outcomes were assessed in week 16 (July 4 - July 10, 2020). Covariates were measured at the beginning of the lockdown (week 1, March 21 - March 27, 2020). The analytic sample was restricted to those who had participated in the religion module (weeks 14-15) and complete the survey in both weeks 1 and 16. Multiple imputation was performed to impute missing data on the covariates and the outcomes.

^b^ All continuous outcomes (life satisfaction, happiness, meaning, social support, loneliness, compliance with social isolation, depressive symptoms, anxiety, and number of minor and major stressors) were standardized (mean = 0, standard deviation, 1), and β was the standardized effect size. The estimates for the outcomes of volunteering, caring, no unhealthy change in smoking behaviors, and thoughts of self-harm were odds ratios estimated via weighted logistic regression; these outcomes were rare (prevalence <10%), so the odds ratios would approximate the risk ratios. The estimates for other dichotomized outcomes (compliance with social distancing, no unhealthy change in drinking, no unhealthy change in diet, gentle physical activity, high intensity physical activity, and good sleep) were risk ratios estimated via weighted Poisson regression.

^c^ All models were controlled for participants’ sociodemographic characteristics (age, gender, race, living alone, education, employment, any key worker role, and low income), health (number of health conditions, current smoking, and number of alcohol drinks in the past week), pre-pandemic religious service attendance, not leaving home, social relationships (frequency of meeting up with people in usual life and number of close friends), personality (neuroticism, extraversion, openness, agreeableness, and conscientiousness), and the prior outcome values wherever data were available. Specifically the adjustment of the prior values were made for the following outcomes simultaneously: psychological well-being (life satisfaction and meaning), social well-being (social support and loneliness), pro-social/altruistic behaviors (volunteering, caring, and compliance with social isolation), psychological distress (depressive symptoms, anxiety, and number of major/minor stressors), health behaviors (change in smoking, change in alcohol drinking, gentle/high intensity physical activity, exercising at home, and sleep). Data were weighted to the proportions of gender, age, ethnicity, education, and country of living obtained from the Office for National Statistics.

Table S5. Comparison of weighted characteristics in week 1 between the initial participants (n=28,847) and the analytic sample (n=8,951)

| Characteristics at the beginning of lockdown | Initial Participants (n=28,847) | | Analytic Sample (n=8,951) | |
| --- | --- | --- | --- | --- |
|  | n (%) | Mean (SD) | n (%) | Mean (SD) |
| Total |  |  |  |  |
| Sociodemographic factors |  |  |  |  |
| Age, years |  | 48 (17) |  | 49 (17) |
| Female gender | 14,501 (51) |  | 4,535 (51) |  |
| Non-white ethnicity | 3,682 (13) |  | 1,144 (13) |  |
| Living alone | 5,170 (18) |  | 1,664 (19) |  |
| Education |  |  |  |  |
| GCSE or below | 9,433 (33) |  | 2,920 (33) |  |
| A levels or equivalent | 9,779 (34) |  | 3,028 (34) |  |
| Degree or above | 9,635 (33) |  | 3,003 (34) |  |
| Employed | 16,604 (58) |  | 4,962 (55) |  |
| Any key worker role | 6,409 (22) |  | 1,868 (21) |  |
| Low Income (<£30 000) | 12,654 (49) |  | 3,881 (49) |  |
| Physical health |  |  |  |  |
| Number of health conditions |  | 0.97 (1.32) |  | 0.92 (1.27) |
| Not leaving home | 15,373 (56) |  | 4,732 (54) |  |
| Social relationships |  |  |  |  |
| Meeting up with people in usual life |  |  |  |  |
| Every day | 3,372 (12) |  | 922 (10) |  |
| Once a week or more often | 15,857 (55) |  | 5,023 (56) |  |
| Less than once a week | 9,618 (33) |  | 3,006 (34) |  |
| Number of close friends |  | 4.5 (3.1) |  | 4.4 (3.2) |
| Personality |  |  |  |  |
| Neuroticism (range: 3-21) |  | 11.5 (4.5) |  | 11.5 (4.5) |
| Extraversion (range: 3-21) |  | 12.6 (4.3) |  | 12.2 (4.3) |
| Openness (range: 3-21) |  | 14.8 (3.4) |  | 14.6 (3.3) |
| Agreeableness (range: 3-21) |  | 15.4 (3.2) |  | 15.4 (3.1) |
| Conscientiousness (range: 3-21) |  | 15.6 (3.1) |  | 15.8 (2.9) |
| Prior psychological well-being |  |  |  |  |
| Life satisfaction (range: 0-10) |  | 5.35 (2.57) |  | 5.51 (2.45) |
| Meaning (range: 0-10) |  | 5.77 (2.74) |  | 5.88 (2.70) |
| Prior social well-being |  |  |  |  |
| Social support (range: 1-30) |  | 22 (6) |  | 22 (6) |
| Loneliness (range: 1-9) |  | 4.96 (1.95) |  | 4.81 (1.91) |
| Pro-social/altruistic behaviors |  |  |  |  |
| Volunteering | 2,004 (7.4) |  | 484 (5.6) |  |
| Caring | 5,817 (21) |  | 1,564 (18) |  |
| Compliance with social isolation (range: 1-7) |  | 6.40 (0.94) |  | 6.43 (0.90) |
| Prior psychological distress |  |  |  |  |
| Depressive symptoms (range: 0-27) |  | 7 (6) |  | 6.1 (6.0) |
| Anxiety (range: 0-21) |  | 6.1 (5.9) |  | 5.6 (5.7) |
| Number of minor stressors (range: 0-16) |  | 4.9 (3.2) |  | 4.7 (3.0) |
| Number of major stressors (range: 0-16) |  | 1.81 (2.29) |  | 1.55 (2.05) |
| Prior health behaviors |  |  |  |  |
| Smoking status |  |  |  |  |
| Current smoker | 4,303 (15) |  | 937 (10) |  |
| Ex-smoker | 7,600 (26) |  | 2,182 (24) |  |
| Non-smoker | 16,944 (59) |  | 5,833 (65) |  |
| Number of alcoholic drinks in the past week |  | 3.7 (5.2) |  | 3.6 (5.1) |
| No unhealthy change in smoking | 26,659 (94) |  | 8,549 (97) |  |
| No unhealthy change in alcohol drinking | 23,763 (84) |  | 7,607 (86) |  |
| Gentle physical activity | 16,863 (62) |  | 5,266 (61) |  |
| High intensity physical activity | 4,222 (16) |  | 1,149 (13) |  |
| Exercising at home | 9,533 (35) |  | 2,852 (33) |  |
| Good sleep | 10,267 (36) |  | 3,348 (37) |  |

Abbreviations: SD, standard deviation

Lockdown was imposed in the UK on March 23, 2020, and we used data from week 1 of the survey (March 21- March 27, 2020). Data were weighted to the proportions of gender, age, ethnicity, education, and country of living obtained from the Office for National Statistics.
